# Gene Model for the ortholog of *Ilp2* in *Drosophila ananassae*

**DOI:** 10.17912/micropub.biology.000917

**Published:** 2026-08-29

**Authors:** Abigail R. Myers, Robyn Huber, Andrew M Arsham, Chinmay P. Rele, Laura Reed

**Affiliations:** 1 The University of Alabama, Tuscaloosa, AL USA; 2 Bemidji State University, Bemidji, MN USA

## Abstract

Gene model for the ortholog of Insulin-like peptide 2 ( *Ilp2* ) in the *D. ananassae* May 2011 (Agencourt dana_caf1/DanaCAF1) Genome Assembly (GenBank Accession: GCA_000005115.1 ) of *Drosophila ananassae* . This ortholog was characterized as part of a developing dataset to study the evolution of the Insulin/insulin-like growth factor signaling pathway (IIS) across the genus *Drosophila* using the Genomics Education Partnership gene annotation protocol for Course-based Undergraduate Research Experiences.

**Figure 1. Genomic neighborhood and gene model for Ilp2 in D. ananassae:** (A) A diagram of synteny of genomic neighborhood of *Ilp2* in *D. melanogaster* and *D. ananassae* . Gene arrows pointing in the same direction as *Ilp2* in both *D. ananassae* and *D. melanogaster* are on the same strand as *Ilp2* ; while gene arrows pointing in the opposite direction are on the opposite strand. The thin underlying arrows pointing to the right indicate that *Ilp2* is on the + strand in *D. melanogaster* ; arrows pointing to the left indicate that *Ilp2* is on the – strand in *D. ananassae* . White arrows in *D. ananassae* indicate the locus ID and the orthology to the corresponding gene in *D. melanogaster* . The gene names given in the *D. ananassae* gene arrows indicate the orthologous gene in *D. melanogaster* , while the locus identifiers are specific to *D. ananassae* . (B) Gene Model in UCSC Track Hub (Raney et al. 2014): the gene model in *D. ananassae* (black), Spaln of *D. melanogaster* Proteins (purple, alignment of refseq proteins from *D. melanogaster* ), BLAT alignments of NCBI RefSeq Genes (blue, alignment of refseq genes for *D. ananassae* ), RNA-Seq from Adult Females (red), Adult Males (blue), RNA-Seq for Wolbachia-cured Embryo (pink), alignment of Illumina RNAseq reads from *D. ananassae* ), and Transcripts (green) including coding regions predicted by TransDecoder and Splice Junctions Predicted by regtools using *D. ananassae* RNA-Seq (Graveley *et al* , 2011; SRP006203 , SRP007906 , PRJNA257286 , PRJNA388952 ). The splice junction shown has a read-depth of 1777 with red supporting splice junctions having a range of >1000. The custom gene model (User Supplied Track) is indicated in black with CDS depicted with wide boxes, intron with narrow lines (arrows indicate direction of transcription). Note that the RNAseq for embryos shows no alignment of expression data at this locus, suggesting that this gene may not be expressed in embryos in this species, which neither supports or refutes the proposed model. Further note that the lack of an aligned Spaln *D. melanogaster* protein at this position indicates that the degree of sequence divergence between the reference gene and the target gene is greater than the minimum similarity needed to see an alignment for this algorithm. By default, Spaln is less sensitive and more specific than BLAST for assigning alignments. (C) Dot Plot of Ilp2-PA in *D. melanogaster* ( *x* -axis) vs. the orthologous peptide in *D. ananassae* ( *y* -axis). Amino acid number is indicated along the left and bottom; while CDS number is indicated along the top and right, and CDSs are also highlighted with alternating background colors. There are two large regions of sequence dissimilarity as displayed by the red (1) and blue (2) boxes. There is also one indel in the middle of CDS two represented by parallel lines. (D) The protein alignment of Ilp2-PA in *D. ananassae* against Ilp2-PA in *D. melanogaster* is shown. Boxes 1 and 2 correspond to the similarly labeled boxes in the Dot Plot highlighting regions of sequence dissimilarity.

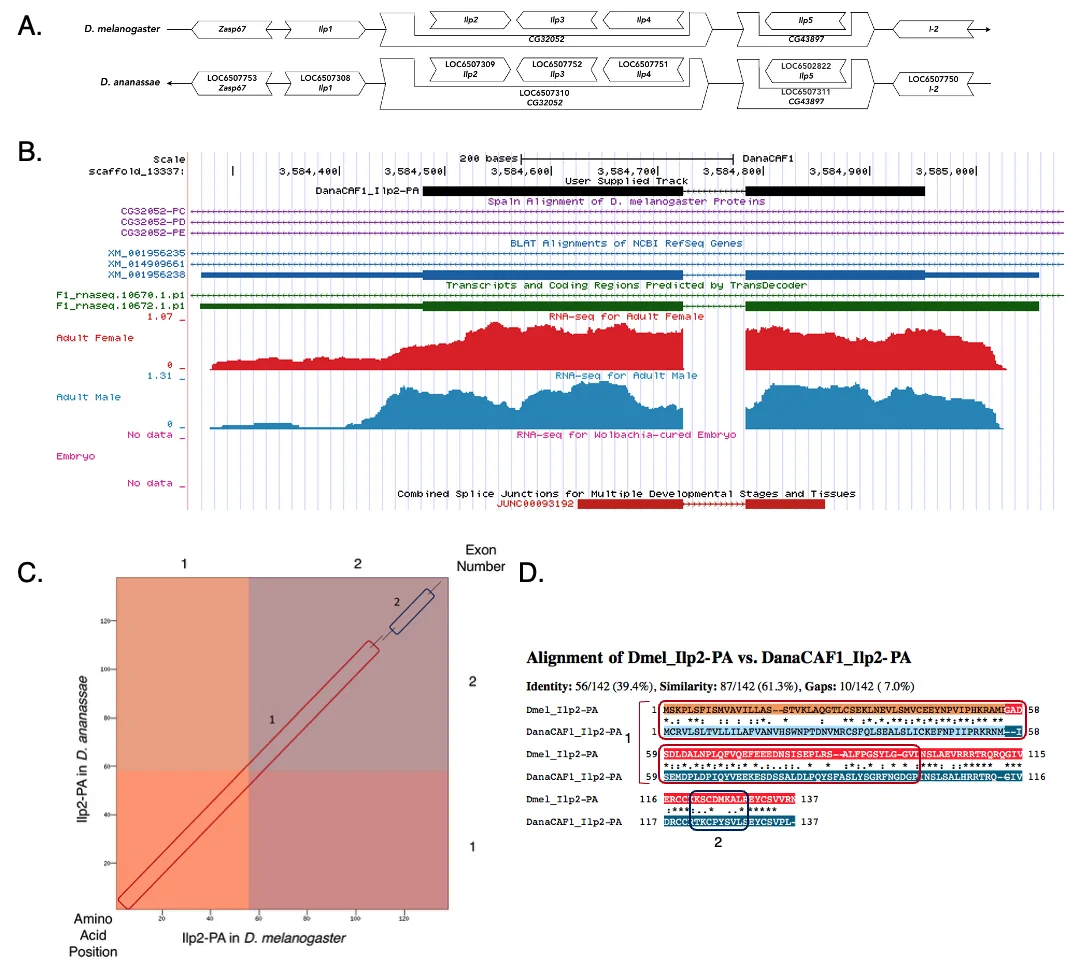



## Description

**Table:** 

*This article reports a predicted gene model generated by undergraduate work using a structured gene model annotation protocol defined by the Genomics Education Partnership (GEP; thegep.org) for Course-based Undergraduate Research Experience (CURE). The following information in this box may be repeated in other articles submitted by participants using the same GEP CURE protocol for annotating Drosophila species orthologs of Drosophila melanogaster genes in the insulin signaling pathway.* "In this GEP CURE protocol students use web-based tools to manually annotate genes in non-model *Drosophila* species based on orthology to genes in the well-annotated model organism fruitfly *Drosophila melanogaster* . The GEP uses web-based tools to allow undergraduates to participate in course-based research by generating manual annotations of genes in non-model species (Rele et al., 2023). Computational-based gene predictions in any organism are often improved by careful manual annotation and curation, allowing for more accurate analyses of gene and genome evolution (Mudge and Harrow 2016; Tello-Ruiz et al., 2019). These models of orthologous genes across species, such as the one presented here, then provide a reliable basis for further evolutionary genomic analyses when made available to the scientific community.” (Myers et al., 2024). “The particular gene ortholog described here was characterized as part of a developing dataset to study the evolution of the Insulin/insulin-like growth factor signaling pathway (IIS) across the genus *Drosophila* . The Insulin/insulin-like growth factor signaling pathway (IIS) is a highly conserved signaling pathway in animals and is central to mediating organismal responses to nutrients (Hietakangas and Cohen 2009; Grewal 2009).” (Myers et al., 2024). “Insulin-like peptide 2 ( *Ilp2* ), a core component of the insulin signaling pathway, mediates growth by acting as a ligand for the Insulin Receptor ( *InR* ) and transducing a signal via the Chico/PI3K/Akt(PKB) pathway (Brogiolo et al., 2001; Park et al., 2014). Ilp2 plays a role in regulating body size by increasing the size and number of cells in individual organs (Ren et al., 2017). Evolutionary studies have shown that loss of *Ilp2* increases lifespan and changes in expression may have contributed to the evolution of body size in the Hawaiian *Drosophila* species (Grönke et al., 2010). In the absence of *Ilp2* , over-expression of *Ilp1* and *Ilp3-7* is enough to promote growth in *Drosophila* (Ikeya et al., 2002). *Ilp2* mutants also seem to have severe developmental delay (Grönke et al., 2010).” (Laskowski et al., 2022). “ *D* . *ananassae* (NCBI:txid7217) is part of the *melanogaster* species group within the subgenus *Sophophora* of the genus *Drosophila* (Sturtevant 1939; Bock and Wheeler 1972). It was first described by Doleschall (1858). *D. ananassae* is circumtropical (Markow and O'Grady 2005; https://www.taxodros.uzh.ch, accessed 1 Feb 2023), and often associated with human settlement (Singh 2010). It has been extensively studied as a model for its cytogenetic and genetic characteristics, and in experimental evolution (Kikkawa 1938; Singh and Yadav 2015).” (Lawson et al., 2024).

The model presented here is the ortholog of *Ilp2* in the May 2011 (Agencourt dana_caf1/DanaCAF1) assembly of *D. ananassae* (Drosophila 12 Genomes Consortium 2007; GCA_000005115.1 ) and corresponds to the Gnomon Peptide ID ( XP_001956274.1 ) predicted model in *D. ananassae* ( LOC6507309 ) *.* This gene model is based on RNA-Seq data from *D. ananassae* (Graveley et al, 2011; SRP006203 , SRP007906 , PRJNA257286 , PRJNA388952 *)* and the *Ilp2* in *D. melanogaster* from FB2022_03 (Larkin et al. *,* 2021; Gramates et al., 2022; Jenkins et al., 2022).

***Synteny***

*Ilp2* occurs on Chromosome 3L in *D. melanogaster* and is flanked by *Zasp67* and *Ilp1* upstream. *Ilp2* is nested in *CG32052* along with *Ilp3* and *Ilp4* to the right. Downstream, *Ilp2* is flanked by *CG43897* (which nests *Ilp5* ) and *I-2* . We determined that the putative ortholog of *Ilp2* is found on scaffold_13337 ( CH902618.1 ) in *D. ananassae* with LOC6507309 (via *tblastn* search with an e-value of 3e-18 and percent identity of 42.70%), where it is flanked by LOC6507753 ( XP_014765450.1 ) and LOC6507308 ( XP_001956275.2 ) which correspond to *Zasp67* and *Ilp1* in *D. melanogaster* with e-values 0.0 and 4e-24 and percent identities 67.67% and 52.46% respectively as determined by *blastp* ( Figure 1A, Altschul et al., 1990). *Ilp2* is nested in LOC6507310 ( XP_001956271.2 ) which corresponds to *CG32052* in *D. melanogaster* with an e-value of 0.0 and a percent identity of 86.67% as determined by *blastp* . Nested in *CG32052* downstream of *Ilp2* are genes LOC6507752 ( XP_001956273.1 ) and LOC6507751 ( XP_032309882.1 ) which correspond to *Ilp3* and *Ilp4* in *D. melanogaster* with e-values of 1e-21 and 7e-28 and percent identities 46.32% and 48.91% respectively, as determined by *blastp* . Downstream of *Ilp2* is LOC6507311 ( XP_044570593.1 ) (which nests LOC6502822 ( XP_001956270.1 ) and LOC6507750 ( XP_001956268.3 ) which correspond to *CG43897 , Ilp5 ,* and *I-2* in *D. melanogaster* with e-values 0.0, 8e-09, and 1e-84 and percent identities 68.85%, 39.51%, and 72.50% respectively, as determined by *blastp.* We suggest this is the correct ortholog assignment for *Ilp2* in *D. ananassae* because local synteny is conserved and although there's a low percent similarity (46.79%) between Ilp2-PA in *D. ananassae* and Ilp2-PA in *D. melanogaster.*

***Protein Model***

*Ilp2* in *D. ananassae* has one protein coding isoform (Ilp2-PA) ( Figure 1B ). Isoform (Ilp2-PA) contains two protein coding CDSs. Similarly, *Ilp2* in *D. melanogaster* has one protein coding isoform (Ilp2-PA) with two coding CDSs *.* The sequence of Ilp2-PA in *D. ananassae* has 46.79% identity with Ilp2-PA in *D. melanogaster* as determined by *blastp* ( Figure 1C ). There are large portions of sequence dissimilarity throughout the gene model as indicated by the red and blue boxes in the Dot Plot ( Figure 1C ). The coordinates of the curated gene models can be found in NCBI at GenBank/BankIt using the accession BK064414 . These data are also available in Extended Data files below, which are archived in CaltechData.

***Special characteristics of the protein model***

**Sequence dissimilarity in gene model:** The regions of sequence dissimilarity are highlighted with boxes in the Dot Plot ( Figure 1C ) and protein alignment ( Figure 1D ). Although these regions appear to be large, Ilp2-PA's overall short coding sequence amplifies the length of the gaps in the Dot Plot.

## Methods

Detailed methods including algorithms, database versions, and citations for the complete annotation process can be found in Rele et al. (2023). Briefly, students use the GEP instance of the UCSC Genome Browser v.435 (https://gander.wustl.edu; Kent WJ et al., 2002; Navarro Gonzalez et al., 2021) to examine the genomic neighborhood of their reference IIS gene in the *D. melanogaster* genome assembly (Aug. 2014; BDGP Release 6 + ISO1 MT/dm6). Students then retrieve the protein sequence for the *D. melanogaster* reference gene for a given isoform and run it using *tblastn* against their target *Drosophila* species genome assembly on the NCBI BLAST server (https://blast.ncbi.nlm.nih.gov/Blast.cgi; Altschul et al., 1990) to identify potential orthologs. To validate the potential ortholog, students compare the local genomic neighborhood of their potential ortholog with the genomic neighborhood of their reference gene in *D. melanogaster* . This local synteny analysis includes at minimum the two upstream and downstream genes relative to their putative ortholog. They also explore other sets of genomic evidence using multiple alignment tracks in the Genome Browser, including BLAT alignments of RefSeq Genes, Spaln alignment of *D. melanogaster* proteins, multiple gene prediction tracks (e.g., GeMoMa, Geneid, Augustus), and modENCODE RNA-Seq from the target species. Detailed explanation of how these lines of genomic evidenced are leveraged by students in gene model development are described in Rele et al. (2023). Genomic structure information (e.g., CDSs, intron-exon number and boundaries, number of isoforms) for the *D. melanogaster* reference gene is retrieved through the Gene Record Finder (https://gander.wustl.edu/~wilson/dmelgenerecord/index.html; Rele et al *.,* 2023). Approximate splice sites within the target gene are determined using *tblastn* using the CDSs from the *D. melanogaste* r reference gene. Coordinates of CDSs are then refined by examining aligned modENCODE RNA-Seq data, and by applying paradigms of molecular biology such as identifying canonical splice site sequences and ensuring the maintenance of an open reading frame across hypothesized splice sites. Students then confirm the biological validity of their target gene model using the Gene Model Checker (https://gander.wustl.edu/~wilson/genechecker/index.html; Rele et al., 2023), which compares the structure and translated sequence from their hypothesized target gene model against the *D. melanogaster* reference gene model. At least two independent models for a gene are generated by students under mentorship of their faculty course instructors. Those models are then reconciled by a third independent researcher mentored by the project leaders to produce the final model. Note: comparison of 5' and 3' UTR sequence information is not included in this GEP CURE protocol (Gruys et al., 2025).
